# Endoscopic embolization of a refractory bronchobiliary fistula by endoscopic retrograde cholangiography using coils and Histoacryl

**DOI:** 10.1055/a-2017-9651

**Published:** 2023-04-27

**Authors:** Paul Didden, Rutger C. G. Bruijnen, Evert-Jan P. A. Vonken, Frank P. Vleggaar

**Affiliations:** 1 University Medical Center Utrecht, Department of Gastroenterology and Hepatology, Utrecht, Netherlands; 2 University Medical Center Utrecht, Department of Radiology, Utrecht, Netherlands

Bronchobiliary fistulae are often related to hepatic tumors and also occur after ablative therapy 1 2 . Endoscopic sphincterotomy or stenting is considered first-line treatment. In case of refractory fistulas, sealing with glue or coils has been suggested, however data are scarce 3 4 .

We present a 53-year-old woman who underwent locoregional treatments of colorectal liver metastasis, including segment 2/3 and wedge resections, radiofrequency ablation (segment 8), and radio-embolization. Plastic stents (after sphincterotomy) were previously inserted to treat sclerosis of both hepatic ducts related to intra-arterial chemotherapy. She was referred for endoscopic management of a refractory bronchobiliary fistula, resulting in bilioptysis.

Endoscopic retrograde cholangiography (ERC) confirmed leakage from a peripheral bile duct in segment 8 towards the bronchial tree ( Video 1 , Fig. 1 ). A 4-Fr tapered Glo-tip catheter (Cook Medical, Bloomington, Indiana, USA) was advanced just underneath the fistula. Two coils (Tornado 0.035 inch, 4–3 mm, and MRey 0.035 inch, 5 mm; Cook Medical) were deployed at the fistula by pushing them through and out the catheter with a standard 0.035-inch guidewire. Two plastic stents were re-inserted over the persistent hepatic duct strictures.

**Video 1** Sealing of a bronchobiliary fistula by endoscopic retrograde cholangiography using coils and Histoacryl.


**Fig. 1:**
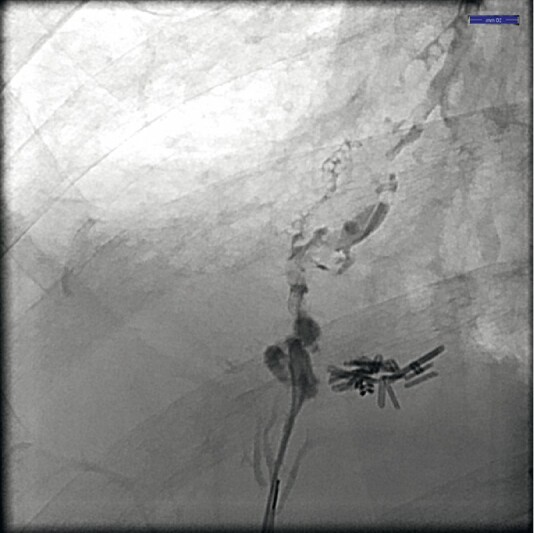
Cholangiography demonstrating bronchobiliary fistula.

After initial resolution, bilioptysis recurred after two weeks and a second ERC was performed showing persistent leakage alongside the coils. A cannula (Tandem XL; Boston Scientific, Marlborough, Massachusetts, USA) was advanced and was flushed with a 5 % glucose solution. N-butyl-2-cyanoacrylate (Histoacryl; B. Braun, Melsungen, Germany) was diluted with Lipiodol (Guerbet GmbH, Sulzbach, Germany) (0.5 ml: 0.5 ml). The glue mixture was injected slowly by pushing it with a 5 % glucose solution. The catheter was pulled back under fluoroscopic guidance during injection. An elongated glue cast was seen after injection of 0.3 cc around the coils and in the end of the peripheral bile duct. Two plastic stents were repositioned ( Fig. 2 ). No complications occurred and the patient remained symptom-free during 2 months’ follow-up.

**Fig. 2:**
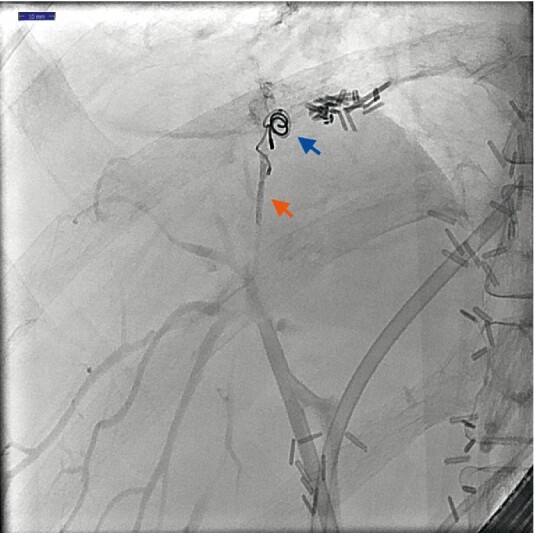
Presence of two coils (blue arrow) and glue cast (red arrow) at the fistula and in the small peripheral bile duct.

Endoscopy_UCTN_Code_TTT_1AR_2AG

## Citation Format

Endoscopy 2023; 55 (S01); E268–E269. doi: 10.1055/a-1974–9202.

## Notes

Endoscopy E-Videos

https://eref.thieme.de/e-videos
*Endoscopy E-Videos* is an open access online section, reporting on interesting cases and new techniques in gastroenterological endoscopy. All papers include a high quality video and all contributions are freely accessible online. Processing charges apply (currently EUR 375), discounts and wavers acc. to HINARI are available. This section has its own submission website at https://mc.manuscriptcentral.com/e-videos
